# COVID-19 in homeless populations: unique challenges and opportunities

**DOI:** 10.2217/fvl-2020-0156

**Published:** 2020-06-26

**Authors:** Brian Conway, David Truong, Kelli Wuerth

**Affiliations:** ^1^Vancouver Infectious Diseases Centre, 201-1200 Burrard St, Vancouver, BC V6Z 2C7, Canada

**Keywords:** COVID-19, emerging outbreak, homelessness, opioid agonist therapy, substance use disorder, unstably housed

At the time of this writing, over five million cases of COVID-19 have been reported worldwide. Quite apart from issues around testing, personal protective equipment and ventilator capacity, several public health interventions are a key part of our response to the pandemic. In our city of Vancouver, the message is clear: stay home, stay put. We are invited to do five things: stay home if we are sick; stay at least 2 meters away from others outside our home; avoid any significant gathering of people; work at home if we can; avoid nonessential outings. These behaviors have led to an unprecedented shift in societal organization and individual behaviors. Vulnerable populations, such as those in urban Vancouver, are both more susceptible to the disease and less able to implement recommended public health efforts, leading to the question of how to address the needs and safety of these populations during a global pandemic.

## Vancouver's Downtown Eastside

Vancouver is home to the Downtown Eastside (DTES), a 50-block area with a population of 18,000 situated east of the city's central business district. The DTES is an intersection of multiple societal issues, including poverty, homelessness, mental health issues and substance misuse. Fifty-three percent of the DTES population is low income, compared with 13.6% of the population of Metro Vancouver [1]. Of the approximately 2200 individuals in Vancouver who are homeless, most live in this neighborhood [2]. Housing is inadequate, mostly consisting of poorly maintained single room occupancy dwellings, with no private cooking or sanitary facilities. A 2013 study of DTES residents in marginal housing found that 95.2% had some form of substance dependence and 47.4% had psychosis [3]. Estimates of the total number of injection drug users in the DTES range from 8000 to 12,000 people [4]. Canada has been in the grips of a national opioid epidemic for years, with the province of British Columbia declaring it a public health emergency in April 2016. Since this declaration, over 1200 opioid-related overdose deaths have occurred in Vancouver alone. Unsurprisingly, the DTES has been particularly affected by the crisis, with fentanyl responsible for the majority of overdoses. The DTES now faces another public health crisis: the COVID-19 pandemic.

## Applying general public health recommendations to homeless populations

On 17 March 2020, the province implemented another public health emergency to address the COVID-19 pandemic. However, recommendations for hand washing, surface disinfecting and physical distancing cannot practically be applied to people without adequate housing. As a result, many locations in the DTES that were the focus of essential daily services for homeless and unstably housed individuals, such as free community or hygiene facilities, closed soon after the declaration. Emergency shelters cut their capacity to meet physical distancing guidelines, increasing the unsheltered population. There was no access to hand washing or hand sanitizing stations. Physical distancing could not be practically enforced at offices distributing social assistance checks. The DTES is home to a very successful supervised injection site, known as InSite, in operation since 2003. As part of the social distancing requirements, it decreased its capacity from 24 to 6 stalls, removing another community safeguard. During the ongoing opioid crisis, key interventions to reduce overdose events have been the widespread distribution of take home naloxone kits in the DTES and encouragement to not use alone, a practice known as ‘buddying.’ In the current situation, buddying must be combined with social distancing (i.e., your buddy must be 2 m or more away). This may be impractical, leading to an increase in the frequency of using alone, a very dangerous practice in a setting where the most commonly available opioids are fentanyl and carfentanil.

Medical care, including opioid agonist therapy, transitioned to delivery by telehealth or electronic portals, modalities often unfamiliar and unsatisfying to this population. At first, testing for SARS-CoV-2 in British Columbia was typically reserved for those presenting at hospitals, but has recently been expanded to include all symptomatic individuals. Despite this increased access, it is likely that COVID-19 is underdiagnosed in the DTES. Some of the symptoms of opioid withdrawal (fever, body aches, tachypnea) overlap with those of COVID-19, suggesting that those with opioid use disorder might not appreciate that a symptomatic infection is present. Many people living in the DTES are also disengaged from the healthcare system, meaning they may not seek out care even if they have COVID-19 symptoms. Finally, there is the issue of asymptomatic infections. At a homeless shelter in Boston, 147/408 (36%) of residents at the shelter tested positive for SARS-CoV-2, yet only 12.2% of those had any symptoms [5]. Similarly, within three shelters in Washington state, 35/195 (18%) of residents and 8/38 (21%) of staff members tested positive, with 86% of those cases identified through systematic testing rather than a symptom-based approached [6].

## Creative efforts to make a square peg fit a round hole

To their credit, public health officials recognized the dilemma and implemented corrective measures. Portable bathroom, shower and laundry facilities were set up, as were hand washing and nonalcohol-based disinfectant stations (the risk of diversion of potentially poisonous alcohol-based products is too high). Some locations that provide services to the homeless began giving out bag lunches. Emergency housing was made available at two community centers away from the DTES. Significant attempts were made to maintain opioid agonist therapy, even if this meant prescription renewals at a distance without a dedicated medical assessment.

Governmental authorities also issued guidance for safer drug use in the COVID-19 era. These guidelines suggest not sharing supplies or equipment. If sharing occurs, all paraphernalia should be wiped down with alcohol. The population is also advised to prepare for a disruption in street drug availability. The Harm Reduction Coalition suggested that one should stock up on injection supplies and drugs and to consider alternative drugs or medications if the drug of choice is not available [7].

Taken together, these measures helped, but could not completely address the issues. Access to showers and laundry is by appointment, and the food offerings have decreased in quality and quantity. Some of the recommendations for drug use during the pandemic may lead to other issues, such as people with a large supply of drugs increasing their consumption or being targeted by other people who use drugs. Unsurprisingly, opioid-related overdose deaths increased by 61% from February 2020 to March 2020 [8].

## Future perspective

Thankfully, there have been remarkably few cases of COVID-19 infection diagnosed to date on the DTES, although this may be due to limited testing. However, if an outbreak were to occur (as has been documented in nearby long-term care facilities or meat packing plants in our city, as examples), it would be extremely hard to contain [5,6].

Essential public health measures cannot be practicably applied for homeless populations, in the DTES and other similar areas. The process of isolation and contact tracing is challenging, and the risk of high-level onward transmission significant. There is an urgent need to re-establish the social networks that have been disrupted to ensure that populations of homeless and unstably housed individuals do not feel further marginalized. There is also an urgent need to restore optimal harm reduction strategies. Combining them with the imperatives of social distancing can be done, especially in a setting where considerable sums of money are available in our societal COVID-19 response.

We propose a three-point plan for action for helping homeless and unstably housed populations during this time (Table 1):

- *Implement new systems to distribute food and provide access to hygiene facilities.* One option is repurposing locations closed due to COVID-19 to meet these needs. This would significantly increase the number of locations where services can be provided and restore the usual level of intervention while allowing for appropriate physical distancing. This is not ‘preferential treatment.’ Natural coping strategies or alternative arrangements to meet these challenges are not available to this population. The alternative is to accept the new imposed order with its lack of physical distancing and higher risk of disease transmission.
- *Improve access to medical care and addiction care.* To limit interactions between patients and providers and reduce the risk of COVID-19 disease transmission, organizations have suggested delivering care with phone or video consults along with increased prescription lengths [9]. Our local guidelines suggest providing month-long opioid prescriptions, even among individuals that were receiving daily dispensed medications and for whom carries have never been attempted. As noted above, we are already seeing a steep increase in opioid-related mortality as a result of these approaches. The solution to this is obvious. Our center and some others have remained open. We have implemented strict physical distancing and reduced the number of patients present in the clinic at any one time. Many pharmacists are willing to dispense medications daily, as long as requirements for observed consumption are relaxed, to limit patient contact time. In this model, other medical needs can be addressed, including abscesses and cellulitis, HCV and HIV infections and psychiatric co-morbidities. Clearly, these strategies would reduce the capacity of the clinics to accommodate the same number of daily patient visits. We propose this could be mitigated by using telehealth or other modes of remote care delivery when they are considered strictly equivalent to a face-to-face interaction, which would remain the preferred method of interaction.
- *Implement strategies to provide long-term housing.* ‘Stay-at-home’ orders are incongruous for people without a home. Lack of housing perpetuates social inequities, favors disengagement from medical and addiction-related care and grossly increases the risk of a massive community-wide COVID-19 outbreak that may well spill over into the broader community. As massive sums are invested and we speak of a slow journey toward a ‘new normal,’ we must take the bold step to address the fundamental issue of housing for this population once and for all. Housing must be safe, comfortable (including individual cooking and sanitary facilities), and integrated into a comprehensive plan of social and healthcare support. Robust and accessible programs of recovery from addiction must also be available. The affected community must be part of the planning and implementation of this transformational program. In Vancouver, as part of the COVID-19 response, community centers and hotel rooms have been earmarked to house those who may need isolation or quarantine. We need to explore how this upgrade in housing can be made permanent. We may never have another opportunity, both logistically and financially, to do this. Lessons learned may well apply to other vulnerable populations, such as refugees and indigenous populations.

**Table 1.** Recommendations for optimal care of homeless populations in the setting of the COVID-19 pandemic.

Problem	Solution
**Disruption of daily life** • Limited access to community centers, food services and sanitary facilities • Reduced social network	Retain or improve the quality of services that were previously available in a way that respects social distancing by increasing the number of service locations while respecting public health imperatives
**Suboptimal healthcare** • Reduced access to healthcare providers • Reduced access to optimal addiction care	Ensure that all clinics and pharmacies are set up to deliver care in person while respecting physical distancing guidelines. Optimized use of telehealth strategies would compensate for the reduced volume of in-person care that could be accommodated
**Housing insecurity** • Reduced access to transitional housing options to avoid homelessness • Much of existing housing inconsistent with requirements of ‘stay at home’ orders	Use funding for the COVID-19 response to address the housing crisis for this population in a generational manner

To our minds, the greatest opportunity of the COVID-19 pandemic is to address the issue of homelessness and unstable housing. This is a factor associated with more problematic drug use, more medically significant drug overdose events and more frequent HIV and HCV infections. It is also one of the key social determinants of health. Secure and stable housing is empowering and will facilitate engagement in care to address the many medical, psychological, social and addiction-related needs of the homeless and unstably housed population. In Vancouver, this includes the dual health crises of the opioid epidemic and COVID-19.

We are told on a daily basis that the COVID-19 pandemic is asking us to act boldly, decisively and as a whole society. Let us not forget the most vulnerable among us. Let us act on a unique opportunity to help them not only weather the storm but come out the other side with renewed hope.
